# Vaccination for the control of childhood bacterial pneumonia — *Haemophilus influenzae* type b and pneumococcal vaccines

**DOI:** 10.15172/pneu.2013.2/229

**Published:** 2016-07-02

**Authors:** Diana C. Otczyk, Allan W. Cripps

**Affiliations:** School of Medicine, Griffith Health Institute, Griffith University, Gold Coast Campus, Queensland, 4222 Australia

**Keywords:** pneumonia, bacteria, *Haemophilus influenzae* type b, *Streptococcus pneumoniae*, vaccine, WHO

## Abstract

Pneumonia in childhood is endemic in large parts of the world and in particular, in developing countries, as well as in many indigenous communities within developed nations. *Haemophilus influenzae* type b and *Streptococcus pneumoniae* conjugate vaccines are currently available against the leading bacterial causes of pneumonia. The use of the vaccines in both industrialised and developing countries have shown a dramatic reduction in the burden of pneumonia and invasive disease in children. However, the greatest threat facing pneumococcal conjugate vaccine effectiveness is serotype replacement. The current vaccines provide serotype-specific, antibody-mediated protection against only a few of the 90+ capsule serotypes. Therefore, there has been a focus in recent years to rapidly advance technologies that will result in broader disease coverage and more affordable vaccines that can be used in developing countries. The next generation of pneumococcal vaccines have advanced to clinical trials.

## 1. Introduction

*Streptococcus pneumoniae* (pneumococcus) and *Haemophilus influenzae* type b (Hib) are the causative pathogens of serious childhood invasive disease, including meningitis, bacteraemia and pneumonia. Both pneumococcus and Hib colonise the nasopharynx — the bacteria being transferred by respiratory droplets. Children younger than 5 years are most susceptible to infection with the risk of infection greatest in children less than 2 years of age. In 2000, *S. pneumoniae* was estimated to be responsible for 14.5 million episodes per year of serious pneumococcal disease in children under 5 years — 96% of those cases were attributable to pneumonia [1] while Hib caused about 8.13 million serious illnesses [2]. Effective vaccines are available to combat pneumonia, however, in poor countries accessibility to vaccines are limited by many factors, including a lack of political support, economic or humanitarian crises, poor or non-existent health service infrastructure and/or existing vaccines being either too expensive or not optimal for developing country use. While other unimmunised children live in indigenous communities in countries that can afford to provide immunisation, however, have not been given priority.

Prior to 2005, the expensive cost of the Hib conjugate vaccine compared to classic childhood vaccines was a major obstacle to prevention of Hib disease. Following the implementation of the Hib Initiative and WHO recommendation that the vaccine be included in routine immunisation programmes of all countries [3], the demand for the vaccine increased [4]. The pneumococcal conjugate vaccine has been available for more tha 10 years and although most of pneumococcal morbidity and deaths occur in Africa and South East Asia, only 15 countries in Africa and none in South East Asia have implemented the vaccine into their national immunisation schedules [5].

This review addresses the current status quo of Hib and pneumococcal conjugate vaccines and their global impact on the disease burden of childhood pneumonia. It also provides an overview of the development of new pneumococcal vaccines and their advancement to clinical studies.

## 2. The role of vaccines in the prevention and control of pneumonia in children

### 2.1. Hib conjugate vaccines

The monovalent Hib conjugate vaccine has been available since the late 1980s and was first licensed in the United States (US) for use in infants in 1990. Hib conjugate vaccines are composed of capsular polysaccharide, polyribosylribitol phosphate (PRP), conjugated to a protein carrier such as diphtheria toxin (D; PRP-D); non-toxic cross-reactive material of diphtheria toxin197, (CRM197; PRP-CRM197); tetanus toxoid (TT; PRP-TT) or meningococcal outer membrane protein (OMP; PRP-OMP). The latter three Hib conjugate vaccines have consistently shown to be effective against invasive disease and are used worldwide. PRP-OMP confers a protective antibody response after the first dose, requires only 2 doses to complete the primary course [6] and is the vaccine of choice in populations with a high incidence of early onset disease. In comparison, PRP-TT achieves protective antibody levels only after the administration of the second dose of the vaccine [7], and 2 or 3 doses of PRP-CRM197 conjugate are required [8]. PRP-D was found to be poorly immunogenic [8] and is no longer used in infants. There are a large number of Hib conjugate vaccine formulations currently available. The monovalent Hib conjugate vaccine can be administered separately or combined with one or more of the following vaccines; hepatitis B (HepB), diphtheria-tetanus-pertussis (DTP), and/or inactivated oral polio vaccines (OPV) to produce tetravalent, pentavalent and hexavalent vaccines. National immunisation schedules for the administration of the Hib conjugate vaccine differ, although in general, WHO recommend a 3-dose primary schedule of 6, 10, 14 weeks given at the same time as DTP plus a booster dose at 12–18 months (3p+1) [3].

Numerous studies have shown Hib conjugate vaccines to be highly effective in preventing serious disease associated with Hib in children under 5 years of age. The Hib conjugate vaccine has provided an 80% reduction in Hib invasive disease (95% confidence interval (CI), 46 to 93%) [9] as determined by a Cochrane meta-analysis of four randomised controlled studies conducted in Chile, Finland, Gambia and Alaska [9]. Significant heterogeneity was observed between the studies, however, the report concluded that the size of the vaccine effects did not appear to be related to the different vaccine types (PRP-CRM197, PRP-TT, PRP-OMP and PRP-D). PRP-D has been withdrawn from production as the new Hib conjugate vaccines were found to be more effective. A meta-analysis of four studies found that the Hib conjugate vaccine prevented 5% of cases of clinical pneumonia (95% CI, 1 to 9%) in The Gambia and Indonesia and 21% of radiologically confirmed pneumonia (95% CI, 3 to 36%) in The Gambia, Indonesia, Bangladesh and Chile [2]. The large between-study variability, however, is a contributing factor to uncertainty in the overall model estimates. Using the Lives Saved Tool (LiST) model to generate an estimate of vaccine effectiveness, a systematic review of cluster randomised controlled trials showed the Hib conjugate vaccine to be 18% effective against radiologically confirmed pneumonia [10].

Herd immunity has played a major role in the impact of the Hib conjugate vaccine worldwide, even at relatively low levels of vaccination coverage — a significant advantage in resource-poor settings. Following PRP-TT introduction in The Gambia, there was virtual elimination of all Hib disease despite only 68% coverage of all 3 doses of the vaccine [11]. Two doses of vaccine were found to provide 94% protection (95% CI, 67 to 100%) with most Gambian children receiving their second dose too late to be protected directly. Presumably, indirect effects of the vaccine had a significant impact on Hib disease, though, since that time there has been a re-appearance of Hib pneumonia and meningitis [12]. The cause of the re-emergence is unclear in the absence of formal surveillance. In the US, 97% of the Navajo population (95% CI, 72 to 99%) receiving at least 1 dose of PRP-OMP were protected against invasive Hib disease [13]. This herd effect is a result of reduced nasopharyngeal colonisation with the organism in vaccinated infants leading to transmission disruption [11, 14].

### 2.2. Pneumococcal vaccines

The four pneumococcal vaccines commercially available, include: three pneumococcal polysaccharide-protein conjugate vaccines; Pfizer Inc.’s (USA) Prevnar®/Prevenar® (PCV-7) and Prevnar 13™/Prevenar 13™ (PCV-13), GlaxoSmithKline Vaccines’ (Belgium) Synflorix™ (PHiD-CV) and the non-conjugated pneumococcal polysaccharide vaccine, Pneumovax® 23 (PPV-23; Merck & Co., Inc., USA).

#### 2.2.1. Pneumococcal conjugate vaccines

PCV-7 contains purified capsular polysaccharide from each of 7 of the 93 known serotypes (4, 6B, 9V, 14, 18C, 19F and 23F) of pneumococcus and was first approved in the US for routine childhood immunisation in 2000. While the serotypes in PCV-7 are responsible for 65–80% of cases of severe paediatric disease in industrialised countries such as North America, Europe and Australia [15], experience showed that significant disease burden still existed in other geographic regions of the world due to serotypes not included in the vaccine. Similar to the currently available PCV-7, the PCV-13 formulation is conjugated to CRM197. The PCV-13 includes PCV-7 serotypes plus serotypes 1, 3, 5, 6A, 7F and 19A. PHiD-CV includes PCV-7 serotypes plus serotypes 1, 5 and 7F and uses an outer membrane protein of non-typeable *H. influenzae* (NTHi), Protein D, as the main carrier protein for most of the serotypes in the vaccine and TT and CRM197 for serotypes 18C and 19F, respectively. PHiD-CV and PCV-13 were first approved in Europe in 2009 and subsequently worldwide with indications varying for countries. In general, PHiD-CV and PCV-13 are indicated for the active immunisation of infants and children from the age of 6 weeks up to 5 years for the prevention of invasive disease, pneumonia and acute otitis media (AOM) caused by the vaccine serotypes of *S. pneumoniae*. Licensure of the two new pneumococcal conjugate vaccines was based predominantly on immunological non-inferiority to PCV-7 and safety. Manufacturers of PHiD-CV and PCV-13 recommend 3 primary doses at 6, 10 and 14 weeks plus a booster between 11 and 15 months (3p+1), or, a 2-dose primary schedule at 2 and 4 months followed by a booster at 10 months of age (2p+1). In 2007, WHO recommended two vaccination schedules for the administration of the pneumococcal conjugate vaccine to infants: a 6, 10, 14 week series (3p+0) and a 2, 4, 6 month series followed by a booster dose at 12–15 months of age (3p+1) [15]. More recently, WHO also recommended the use of a schedule consisting of 2 primary doses as early as 6 weeks of age with a booster dose at 9–15 months of age (2p+1) [16]. Factors relevant to local conditions should be considered when countries are choosing between PHiD-CV and PCV-13 and the dosing schedules to be used. Consequently, pneumococcal conjugate vaccine schedules in use in national immunisation schemes vary according to the number of doses given, the time between doses and the ages at dosing.

The association of particular serotypes with disease may be influenced by several factors, including patient age, predisposition to disease, disease severity, invasive potential, geography and temporal change. Based on the WHO commissioned 2007 Pneumococcal Global Serotype Project [17], 7 serotypes (1, 5, 6A/6B, 14, 19F, and 23F) were found to account for at least 60% of invasive disease worldwide. The 3 most prevalent serotypes are 1, 5 and 14 with serotypes 1 and 5 of significant importance in Africa and parts of Asia. It is anticipated that with the selection of the new serotypes included in PHiD-CV and PCV-13 there will be ≥70% coverage of invasive pneumococcal pneumonia [16]. Notwithstanding, there remain a significant number of unanswered questions regarding the long-term impact of pneumococcal conjugate vaccines on pneumonia morbidity with the emergence of non-vaccine serotype replacement and the spread of antibiotic resistance. Surveillance data from North America, Europe, Australia [18], England and Wales [18, 19] on the incidence of invasive pneumococcal disease following the introduction of PCV-7 have shown varied results. Further to a Strategic Advisory Group of Experts (SAGE) on Immunisation review of surveillance data of serotype replacement following the introduction of PCV-7 [20], WHO concluded that Australia, Canada, England, Wales, South Africa and the USA showed a decline in invasive pneumococcal pneumonia due to vaccine serotypes in children younger than 5 years [16]. Moreover, PCV-7 also showed herd protection in older age groups. There was also evidence of increases in non-vaccine invasive disease among hospitalised cases in some settings in all age groups. Pneumococcal invasive disease caused by all serotypes had declined in children younger than 5 years with mixed results for older age groups in different settings. Importantly, the analysis concluded that “great caution needs to be exercised in interpreting pneumococcal surveillance data” as factors unrelated to the vaccine and surveillance artefacts may potentially bias surveillance outcomes. PCV-7 non-vaccine serotypes 1, 3, 6A, 7F and 19A have become increasingly important causes of invasive pneumonia in children [21–27], in particular, multidrug resistant serotype 19A [28]. PHID-CV protection against serotypes 19A and 6B will rely on cross-protective antibodies induced against 19F and 6A. Several countries, including the US, United Kingdom (UK) and Australia, have transitioned from the use of PCV-7 to use of PCV-13 for the routine vaccination of children. Vigilant surveillance will be required to monitor the impact of the vaccines on serotype prevalence and antibiotic resistance — WHO recommending surveillance at least 2 years prior to and 5 years post vaccine introduction [16].

Use of PCV-7 in the US has demonstrated vaccine efficacy of 97.4% (95% CI, 82.7 to 99.9%) against invasive pneumococcal disease from serotypes included in the vaccine and 89.1% (95% CI, 73.7 to 95.8%) efficacy against disease from all serotypes [29]. An investigational 9-valent pneumococcal conjugate vaccine administered to a population of children in The Gambia decreased the rate of invasive disease from serotypes included in the vaccine by 77% (95% CI, 51 to 90%) and 50% (95% CI, 21 to 69%) for all serotypes [30]. In South Africa, the vaccine efficacy was 20% (95% CI, 2 to 35%) against radiologically confirmed pneumonia [31].

In the US following the introduction of PCV-7 a considerable reduction was observed in invasive pneumococcal disease among children younger than 5 years of age, dropping from 97 to 24 cases per 100,000 population during the period 1998–1999 to 2005 while disease caused by vaccine-type strains fell from 80 to 4.6 cases per 100,000 [32]. Hospitalisation rates for all-cause pneumonia in young children declined by almost 40% [33]. Similar to the US experience, there have been demonstrated reductions in pneumonia following the incorporation of PCV-7 into national immunisation schedules of other countries [34–37]. The LiST estimate of the effectiveness of the pneumococcal conjugate vaccine on radiologically confirmed pneumonia was 26% [10]. The effectiveness of the additional 6 serotypes in PCV-13 in reducing invasive pneumococcal disease in England and Wales has been assessed following its replacement of PCV-7 in the national immunisation schedule [38]. Vaccine effectiveness was 78% (95% CI, −18% to 96%) for 2 doses in children < 12 months and 77% (95% CI, 38 to 91%) for 1 dose in children > 12 months. For those children who received one or more doses of PCV-13, vaccine effectiveness for serotypes 7F and 19A was 76% (95% CI, 21 to 93%) and 70% (95% CI, 10 to 90%), respectively. Vaccine effectiveness for serotypes 1 and 3 was 62% (95% CI, −112 to 92%) and 66% (95% CI, −17 to 90%), respectively. During the 16 month study period, the number of invasive pneumococcal cases in children younger than 2 years had been reduced by 50% due to PCV-13-only serotypes. A cluster-randomised trial in the Netherlands demonstrated PHiD-CV effectiveness of 92% (95% CI, 58 to 100%) to 100% (95% CI, 83 to 100%) against invasive pneumococcal disease in infants using a 2p+1 and 3p+1 schedule, respectively [39].

PCV-7 has had a substantial indirect impact on reducing invasive pneumococcal disease in children under 2 years of age, as well as among infants that were partially immunised [40], as a result of reduced nasopharyngeal carriage and transmission of vaccine serotypes [41]. The measure of indirect protection on non-invasive pneumococcal pneumonia has not been as transparent [42], although, pneumonia hospital admissions in the US in the pre- and post-vaccine era have shown a statistically significant decrease in rates of all-cause pneumonia admissions among young adults (26%; 18 to 39 years) [33] and a 54% reduction (95% CI, 53 to 56%) in nonbacteremic pneumococcal pneumonia in adults ≥ 65 years of age [43]. A recent randomised study in the Netherlands demonstrated that PHiD-CV had no effect on nasopharyngeal NTHi colonisation or acquisition in infants making it unlikely to have any impact on herd protection for NTHi [44].

#### 2.2.2. PPV-23

PPV-23 has been available in the US for nearly 25 years. PPV-23 contains 23 pneumococcal polysaccharide capsular serotypes 1, 2, 3, 4, 5, 6B, 7F, 8, 9N, 9V, 10A, 11A, 12F, 14, 15B, 17F, 18C, 19A, 19F, 20, 22F, 23F and 33F. These serotypes accounted for 85–90% of invasive pneumococcal disease in the industrialised world [45, 46]. PPV-23 is widely licensed for use in adults and children aged ≥ 2 years who have certain underlying medical conditions.

The widespread use of PPV-23 has been restricted because conjugate vaccines were found to have several advantages over PPV-23, including: the induction of protective immunity in children under 2 years of age and a reduction in pneumococcal nasopharyngeal carriage [41]. Nevertheless, there is ongoing debate about the use of PPV-23 in young children [47, 48]. The challenge to improve the serotype coverage, flexibility and affordability of pneumococcal vaccines for resource-poor countries has encouraged ongoing investigations to optimise immunisation schedules with pneumococcal polysaccharide vaccines or in combination with PCV-7.

Three double-blind, placebo-controlled trials in Papua New Guinea in the 1980s evaluated the efficacy of pneumococcal polysaccharide vaccines in young children [49]. Overall, the studies demonstrated a reduction in pneumonia mortality in children by at least 50% when vaccinated at 6 months to 5 years of age with 1 or 2 doses of either 14-valent pneumococcal polysaccharide vaccine or PPV-23 (95% CI, 1 to 75%). A recent meta-regression analysis of the immunogenicity of pneumococcal polysaccharide vaccines in healthy young children showed that the immune response to different vaccine serotypes progressively matured with age and the magnitude of the antibody response was dependent on the geographical location; children in resource-poor countries mounting higher responses suggesting that natural exposure to circulating serotypes primes the immune response [50]. The immunogenicity of PPV-23 for seven serotypes that are frequently responsible for causing pneumococcal invasive disease was evaluated in children from the highlands of Papua New Guinea who had been vaccinated between the ages of 3 months and 5 years [51]. A two-fold or greater increase in serotype-specific IgG antibody levels was observed to serotypes 2 and 7F by 6 months of age, serotypes 23F and 5 before the age of 1 year with a gradual increase in response to serotypes 6B, 14, and 19F up to 2 years of age.

Vaccination of Fijian infants at 6 weeks with a single dose of PCV-7 followed by a PPV-23 booster at 12 months elicited robust responses for the 7 serotypes in common with PCV-7 with significant serotype-specific IgG antibody levels (p<0.001) to the 16 serotypes not included in PCV-7 following PPV-23 immunisation [52]. The PPV-23 booster also enhanced affinity maturation with an increase in antibody avidity for most serotypes [53] However, immunological hyporesponsiveness was observed by reduced responses to all 23 serotypes following a low dose re-challenge of PPV-23 at 17 months [54]. A study in a high-risk population demonstrated that Australian Indigenous children immunised with PCV-7 at 2, 4 and 6 months of age, then boosted with PPV-23 at 18 months were capable of mounting protective levels of IgG antibody against all seven PCV-7 serotypes 1 month after the third dose [55]. One month following PPV-23 immunisation, antibody levels were significantly higher for 15 of the 16 non-PCV-7 serotypes (the exception being serotype 19A) and more than 96% of children had protective levels of antibody for all seven PCV-7 serotypes. Pneumococcal polysaccharide vaccines have failed to have an impact on nasopharyngeal carriage [56–58], with the exception of a study conducted in the 1970s that showed a reduction in carriage in South African miners who had been protected from pneumonia following immunisation with investigational 6-valent and 13-valent vaccines [59].

The strategy of maternal immunisation with pneumococcal polysaccharide vaccine has been investigated as an approach to protect young infants from pneumococcal disease in the vulnerable period. Immunisation during the third trimester of pregnancy with pneumococcal polysaccharide vaccine has demonstrated significant levels of serotype-specific mucosal IgA in the breastmilk of Gambian women 6 months after delivery (p<0.05) [60]. In a small study in Papua New Guinea, immunisation of pregnant women whose babies were 1–17 months of age reduced pneumonia in the infants by 32% during the next 1–5 months (p=0.003), presumably because of increased antibody concentrations in breast milk [61–63]. No immunological suppression was observed in infants to subsequent PPV-23 vaccination [61].

## 3. The role of vaccines in the prevention of pneumonia in high-risk children

Prior to routine infant vaccination, Indigenous communities in developed countries such as Alaskan Native children [64] and Australian Indigenous children [65] experienced extremely high rates of pneumonia; more than 10-fold higher than their non-Indigenous counterparts. Similar to other settings in the world where there is a high pneumonia disease burden, Australian Indigenous children poor health outcomes are associated with socioeconomic circumstances such as poor nutrition, higher and earlier colonisation rates of respiratory pathogens [66], early onset of pneumonia [67] and inadequate health services [68].

Since 2001, Australian Indigenous children have been immunised with a 3-dose PCV-7 schedule in infancy followed by a PPV-23 booster in the second year of life for increased serotype coverage. Non-Indigenous children have used a 3-dose PCV-7 schedule since 2005. Following the introduction of pneumococcal vaccination, the incidence of invasive pneumococcal disease in Indigenous children has decreased, primarily as a result of a reduction in disease caused by PCV-7 serotypes [69, 70] with a corresponding 28 to 44% decline in pneumococcal pneumonia hospitalisations [71]. Though, in some remote Indigenous communities there have been reports of no change in the incidence of radiologically confirmed pneumonia [67] and an increased risk of pneumonia in Indigenous infants following PPV-23 vaccination [72]. Unlike their non-Indigenous counterparts, Indigenous children have not experienced a significant increase in replacement disease due to serotype 19A [69, 70]. Similar to the worldwide experience, however, there have been observed shifts in serotype-specific carriage in Indigenous children following pneumococcal vaccination with 19A, 6A, 6B and 16F being the predominant serotypes [73–75]. Vigilant surveillance is necessitated in this high-risk population if lessons are to be learnt from the alarming increase in serotype replacement disease observed in Alaskan Native children [76, 77]. In 2009, the Northern Territory, Australia, included PHiD-CV for routine immunisation use instead of the combination PCV-7/PPV-23 schedule to protect against the high carriage rate of NTHi in Indigenous children in remote communities and its association with the causation of AOM. In 2011, PCV-13 (3p+0 schedule) replaced PCV-7 and PHiD-CV in the Northern Territory. A booster dose of PCV-13 was recommended at 12–18 months of age for Indigenous children in areas of high incidence, replacing the PPV-23 booster.

Australian Indigenous children experienced the highest incidence of invasive Hib disease prior to the introduction of the Hib conjugate vaccine (PRP-OMP) in national immunisation schedules in 1993 [78]. Since that time there has been a dramatic and sustained decrease in the incidence of Hib disease among Indigenous children. Notifications of invasive Hib disease among Indigenous children dropped from 35 cases per 100,000 population in 1993–1994 to 5 cases per 100,000 population in 2004–2005 [79]. However, the Indigenous population continue to remain at greater risk of invasive Hib disease compared to non-Indigenous Australians. The rate ratios in this population range from 2.7 in 1993–1994 (95% CI, 1.84 to 3.76) to 7.5 in 2004–2005 (95% CI, 2.25 to 19.70), with the highest rate ratio 17.6 in 2002–2003 (95% CI, 8.90 to 32.96) [79]. Following shortages in vaccine supply, there was a transition period between 2005 and 2009 when PRP-OMP was replaced with PRP-TT. Of concern, is the possibility of the re-emergence of invasive Hib disease as experienced in Alaska under similar circumstances [80] and given the continuing high carriage rates of Hib (3.4%) in well vaccinated Indigenous children [81].

## 4. The future direction of pneumococcal vaccines

Currently, Merck & Co. Inc. are conducting a phase II (NCT01215188) study on a 15-valent pneumococcal polysaccharide-protein conjugate vaccine which comprises of the 13 serotypes present in PCV-13 and serotypes 22F and 33F conjugated to CRM197 (PCV-15) [82, 83]. A phase I (NCT01215175) comparator study in adults and toddlers showed that a single dose of PCV-15 had an acceptable safety profile and induced IgG responses to all serotypes included in the vaccine [84, 85]. Antibody levels were comparable between PCV-15 and PCV-7 for the common serotypes. Whilst this early clinical data looks promising for the vaccine candidate, this form of technology will have its limitations in the future as challenges related to limited serotype coverage, serotype replacement and the high cost associated with complex manufacturing processes will make it difficult for developing countries to afford them without external assistance. For these reasons, there is stimulated interest in a new generation of pneumococcal vaccines that have the potential to provide broad, affordable coverage against pneumococcal disease.

Common protein vaccines that contain proteins that are conserved on all pneumococcus serotypes, a combination protein-conjugate vaccine and whole cell vaccines are currently being investigated (Table 1). Numerous pneumococcal proteins have been tested in animal studies [86], however, only a few have progressed to clinical trials. An earlier human study [87] showed the pneumococcal surface protein A (PspA), a cross-reactive protein expressed by all pneumococci, to be immunogenic. Sera from the immunised subjects protected mice from fatal *S. pneumoniae* challenge [88]. In mouse models, PspA has been shown to compete with C-Reactive Protein (CRP) binding to the pneumococcal surface [89] and consequently decreasing the amount of CRP-mediated complement deposited. Furthermore, antibody to PspA can enhance complement deposition [90] and thus enhance the phagocytosis of pneumococci [91]. Immunisation with PspA has also been shown to confer protection against pneumococcal carriage in mice [92], however, protection has yet to be shown in humans. In light of the propensity for the pneumococcus to rapidly adapt to evade immunity induced by clinical intervention [28, 93] and in an attempt to gain greater potency, there has been an emphasis to develop candidate vaccines with more than one common protein.

**Table 1:** Investigational pneumococcal vaccines assessed in clinical trials

Vaccine formulation	Antigens of interest	Clinical trial phase	Vaccination route	Study outcome	Collaborators	Reference or source
Polysaccharide-protein conjugate	1, 3, 4, 5, 6A, 6B, 7F, 9V, 14, 18C, 19A, 19F, 23F, 22F and 33F	II	injected	Adults: tolerated & immunogenic. Toddlers: tolerated & immunogenic. Infants: ongoing tolerability & immunogenicity.	Merck & Co. Inc.	[84, 85] NCT01215175 NCT01215188
Protein-based sub-unit	PspA	I	injected	Adults: tolerated & immunogenic.	Aventis Pasteur	[87]
	PcsB, StkP, PsaA	I	injected	Adults: tolerated & immunogenic.	Intercell AG, PATH	[108] NCT00873431
	PhtD, PcpA, PlyD1, PcpA/PhtD	I	injected	Adults: tolerated and induced functional PlyD1 antibody. Functional post-immune human anti-PhtD antibody. Bangladeshi adults, toddlers & infants: ongoing tolerability & immunogenicity.	Sanofi Pasteur, Covance Clinical Research Unit	[101, 102, 107] NCT01444352 NCT01444001 NCT01444339
Protein-conjugate	dPly, PhtD/dPly, dPly/PHiD-CV, PhtD/dPly/PHiD-CV	II	injected	Toddlers: tolerated and immunogenic. Gambian infants & toddlers: ongoing immunogenicity & carriage.	GSK Biologicals, PATH, The London School of Hygiene and Tropical Medicine, Medical Research Council Unit	[96, 97, 98] NCT00707798 NCT00896064 NCT00985751 NCT01262872
Whole cell	Killed, non-encapsulated *S. pneumoniae*	I	injected	Adults: ongoing safety & immunogenicity.	PATH, Children’s Hospital Boston	NCT01537185
	Live, avirulent PspA-expressing *Salmonella enterica* serovar Typhimurium	I	oral	Adults: ongoing safety & immunogenicity.	Arizona State University	NCT01033409

Abbreviations: PspA, pneumococcal surface protein A; PcsB, putative hydrolase involved in cell wall metabolism and division of Group B Streptococcus; StkP, serine/threonine protein kinase; PsaA, pneumococcal surface adhesion A; PhtD, histadine triad protein; PcpA cell surface choline binding protein; PlyD1, highly detoxified pneumolysin; dPly, detoxified pneumolysin; PHiD-CV, 10-valent pneumococcal nontypeable *Haemophilus influenzae* Protein D conjugate vaccine.

The most advanced protein-based pneumococcal vaccine candidate combines a histadine triad (Pht) protein (PhtD) and detoxified pneumolysin (dPly) with the conjugated polysaccharides in GSK Biologicals’, PHiD-CV. Preclinical evaluations demonstrated that PhtD and human anti-PhtD antibodies protects against *S. pneumoniae*, including serotype 3, in an intranasal lethal challenge mouse model [94]. Furthermore, in a rhesus macaque model of pneumonia, adjuvanted PhtD/dPly induced antibodies that protected monkeys after challenge with *S. pneumoniae* [95]. A phase I (NCT00707798) study assessed various formulations (dPly or dPly/PhtD combined/not combined with PHiD-CV), antigen doses and adjuvantation for safety and reactogenicity in healthy adults compared with a single dose of PPV-23 [96]. A follow-up phase II (NCT00896064) study investigated a third dose of one of two formulations administered 5–9 months after the primary dosing series. A 2-dose primary series of all formulations followed by a booster dose were found to be well tolerated with no serious adverse events. Several investigational vaccine formulations (dPly/PhtD combined/not combined with PHiD-CV) were compared with PHiD-CV and found to be well tolerated and immunogenic in Czech toddlers when administered in a 2-dose primary series plus booster phase II (NCT00985751) study [97]. The vaccine candidate is currently being trialled for safety, immunogenicity and impact on nasopharyngeal carriage in The Gambia as part of the *Streptococcus pneumoniae* immunisation and carriage (SPICAR) study: a collaboration of PATH, The London School of Hygiene and Tropical Medicine (UK), Medical Research Council Unit (Fajara, The Gambia) and GSK Biologicals (Global Vaccine Development, Belgium). The ongoing phase II (NCT01262872) study will assess two formulations of the investigational vaccine with comparators PHiD-CV and PCV-13 in 2–4 year old children and in infants 8–10 weeks of age [98].

Sanofi Pasteur (USA) and Covance Clinical Research Unit AG (Switzerland) have conducted clinical studies designed to assess the safety and immunogencity of the candidate subunit vaccines: PhtD, the choline-binding protein A (PcpA) and a highly detoxified pneumolysin mutant (dPlyD1). Supported by preclinical studies in mice [99, 100], a phase I (NCT01444352) study investigating a 2-dose primary series vaccination with adjuvanted dPlyD1 in healthy adults was found to be well tolerated inducing functional anti-PlyD1 IgG in a dose-related manner that inhibited the toxic activity of pneumococcal pneumolysin to Vero cells [101]. A phase I (NCT01444001) study showed that 3 doses of the PhtD vaccine candidate administered as 2 injections in adults was safe and immunogenic [102]. Anti-PhtD IgG levels showed a dose-related increase. Post-immune human anti-PhtD antibody protected mice against lethal pneumococcal challenge [103]. Ongoing characterisation studies with pneumococcal proteins have shown that PhtD, PhtE and PcpA mediate adherence and human antibody against the proteins can function to prevent adherence to human airway epithelial cells, in vitro [104, 105]. Furthermore, the proteins are able to induce high IgG antibody levels in children (6 to 30 months old) in response to natural infection [106]. Adjuvanted and unadjuvanted vaccine candidates, PcpA and PcpA/PhtD, were found to be safe and immunogenic in a dose-related manner in healthy adults (NCT01444339) [107]. Adjuvantation of the vaccine candidates did not enhance immune responses. A phase I formulation, dosing and adjuvantation (NCT01446926) study in Bangladesh is currently evaluating the safety, tolerability and immunogenicity of investigational protein-based pneumococcal vaccines in healthy adults, 6–7 week infants and 12–13 month toddlers.

IC47 (Intercell AG, Austria), a multivalent recombinant subunit protein vaccine containing the highly conserved proteins: pneumococcal surface adhesion A (PsaA); serine/threonine protein kinase (StkP; a cell division protein which is also involved in signalling) and a putative murine hydrolase (PcsB; involved in cell wall metabolism and division of group B streptococcus) has advanced to a phase I (NCT00873431) study in Germany. Two different dosages of the vaccine candidate, adjuvanted and unadjuvanted, showed the vaccine to be safe and induced antibodies in a dose-dependent manner for all three proteins included in the vaccine in healthy adults (unpublished data) [108]. Characterisation studies have demonstrated that the pneumococcal proteins are naturally immunogenic in unvaccinated adults inducing a Th17 response [109].

PATH and Children’s Hospital Boston (MA, USA) are working on a killed, non-encapsulated whole cell *S. pneumoniae* vaccine candidate to deliver a large number of antigens and potentially induce broad protection against different serotypes. The *S. pneumoniae* strain has been genetically modified to be autolysin-deficient and express nontoxic pneumolysoid rather than pneumolysin. In mice studies, intranasal vaccination with the vaccine candidate affords multi-serotype, antibody-independent protection against pneumococcal nasopharyngeal colonisation via CD4+ Th17 cell-dependent, IL-17A-mediated mechanism [110, 111] while parenteral vaccination induces both antibody that protects against invasive disease, as well as, IL-17A-mediated nasopharyngeal clearance [112] which is serotype independent [113]. When challenged with the whole cell antigen, Th17 cells isolated from the sera of Bangladeshi and Swedish children and adults were found to be specific for pneumococcus and produced differing levels of IL-17A responses [114]. IL-17A responses in Swedish children were low in contrast to robust responses in Bangladeshi children which was comparable to that of Bangladeshi adults, suggesting that differences in natural exposure to pneumococci may play a role. The observation that the whole killed cells can induce both humoral and Th17 cell-mediated immunity has forwarded the candidate vaccine, in an adjuvanted formulation, to be assessed in a dose-escalation phase I (NCT01537185) study for safety and humoral immune response in healthy adults following a 3-dose intramuscular injection schedule.

Numerous studies have investigated the mucosal administration of vaccines as an attractive immunisation strategy to potentially induce both mucosal and systemic protection [115] and has been the strategy of choice for immunisation studies with a live avirulent *Salmonella enterica* serovar Typhimurium vector expressing pneumococcal proteins PsaA and PspA. Preclinical studies in mice have demonstrated that the investigational pneumococcal vaccines when delivered mucosally, confer protection against pneumonia and nasopharyngeal colonisation eliciting both serum IgG and mucosal IgA antibodies [116–118]. A phase I (NCT01033409) study is currently evaluating the safety and immunogenicity of three formulations of recombinant attenuated *Salmonella enterica* serovar Typhimurium strain expressing PspA orally administered to healthy adults.

## 5. Conclusion

There is little doubt that the life-saving Hib and pneumococcal conjugate vaccines have had a significant influence on controlling pneumonia. And yet, despite these very convincing achievements, too many children are still not receiving the required vaccines today. It is anticipated that with accumulating evidence regarding vaccine effectiveness, herd immunity and surveillance assessments on the impact of pneumococcal conjugate vaccines on invasive disease, more countries will be motivated to include the vaccines into national immunisation schedules.

New opportunities are available to overcome the shortfalls of the current pneumococcal conjugate vaccines in the form of a new generation of pneumococcal vaccines that are potentially more affordable and designed to maximise protection in developing countries. These vaccines are still in their early phases of clinical investigation and there will be new challenges ahead in establishing their equivalence or superiority to the currently available polysaccharide conjugate vaccines. There is the promise that global immunisation will continue to improve child survival and significantly reduce the burden of co-morbidities associated with pneumonia.
